# The Danger Is Growing! A New Paradigm for Immune System Activation and Peripheral Tolerance

**DOI:** 10.1371/journal.pone.0008112

**Published:** 2009-12-02

**Authors:** Sharon Bewick, Ruoting Yang, Mingjun Zhang

**Affiliations:** Mechanical, Aerospace and Biomedical Engineering Department, University of Tennessee Knoxville, Knoxville, Tennessee, United States of America; New York University School of Medicine, United States of America

## Abstract

Successful immune defense is a complex balancing act. In order to protect a host against invasion by harmful pathogens, an immune response must be rapid and vigorous, and must eliminate foreign invaders before their populations grow beyond control. That same immune response, however, must be selective enough to recognize and ignore commensal bacteria, environmental antigens and host tissue itself. How the immune system makes the crucial decision whether or not to attack a particular antigen has been a long-standing question central to the study of immunology. Here we show that the structure of the signaling network between regulatory T-cells and type 17 helper T-cells allows the immune system to selectively attack pathogens based on whether or not the pathogens represent a growing, and thus dangerous population. We term this mechanism for immune system activation the ‘Growth Detection Paradigm’, because it offers an entirely new explanation for immune system regulation and peripheral tolerance.

## Introduction

For many years, immunologists have focused on the ability of the immune system to distinguish between non-threatening antigens [1](both foreign and self) and hostile invaders based on pattern recognition. This has lead to intense scrutiny aimed at mapping different pattern recognition receptors (PRRs) to the chemical structures that they recognize, and the classes of microorganisms that stimulate them. Since the discovery of Toll-like receptors (TLRs) in the late nineties [2], for instance, TLRs have come to the forefront of immunological research. We now know that TLRs recognize everything from glycoproteins [3], [4] to liposaccharides [5] and nucleic acids [6], [7], [8], [9], and that, when activated, they can trigger a rapid immune response. Analysis of the adaptive immune system has similarly focused on the ability of T-cell receptors (TCRs) and B-cell receptors (BCRs) to recognize and react to short sequences of amino-acids, or epitopes, characteristic of a stimulating antigen.

TLRs, TCRs and BCRs all recognize spatial patterns. Immunologists, however, in their eagerness to fully understand the multitude of different chemical sequences and structures that the vertebrate immune system can detect, have overlooked the fact that pattern recognition need not be limited to space. Indeed, the large majority of pathogen invaders are characterized by temporal patterns as well and, in many ways, these temporal patterns are less variable than their spatial counterparts when compared across large classes of different microorganisms. In order to survive, all microorganism populations must grow. Certainly, rates of growth can be different, as can periods of latency and steady state population levels. Still, any infectious agent that poses a threat to its host will exhibit the temporal pattern of growth.

By constructing a dynamics model that integrates the known signaling and maturation kinetics of regulatory T-cells (T_reg_), and type 17 helper T-cells (Th17), we show how immune regulation emerges through the encoding of time-dependent information into T-cell population sizes. We then show how the immune system uses this information to decide whether or not an antigen is part of a growing pathogen population. More specifically, the temporal pattern of the antigenic signal leads the immune system to develop either a large excess of T_reg_ cells, in which case peripheral tolerance develops, or else a large excess of Th17 cells, in which case a defensive attack is mounted. We term this mechanism for immune system regulation the ‘Growth Detection Paradigm’, or GDP, and suggest that it can rationalize many of the confusing aspects of T-cell interactions and immune system regulation. In particular, GDP offers a novel explanation for the development of peripheral tolerance, which is the ‘real-time’ ability of the immune system to determine quickly and accurately whether or not a foreign substance is dangerous without having had previous exposure to that antigen. Since peripheral tolerance is one of the most important, yet most poorly understood phenomena associated with immune operation, the GDP interpretation will have profound implications in terms of both interpreting the immune system at a fundamental level and developing improved clinical practices ranging from novel vaccination schemes to treatments for chronic infection and autoimmune diseases.

## Methods

The Growth Detection Paradigm (GDP) is formulated in reference to the kinetics and signaling interactions in the T_reg_/Th17 system. Very generally, after being stimulated by contact with antigens displayed on the surfaces of antigen presenting cells (APCs), certain naïve T cells develop into induced (peripheral) T_reg_ cells, which have the ability to suppress an immune response [10], while others develop into Th17 cells which function as instigators of inflammation [11], autoimmunity [12] and pathogen defense [13]. With respect to building a dynamics model, two empirical observations regarding Th17 and T_reg_ maturation are of particular importance. First, using a lymphopenic mouse model, the Abbas group has shown that Th17 cells mature rapidly compared to peripheral T_reg_ cells in response to antigenic stimulation [14], [15]. Second, it has been shown that a low concentration of TGF-β is required for Th17 cell survival and/or maturation [16], [17], [18], [19] and that the primary source of this cytokine is the mature T_reg_ cell population [20], [21], [22].

Once T_reg_ cells and Th17 cells have matured, they secrete cytokines that stimulate expansion of their own cell populations (positive feedback) [18], [20], [23], [24], while at the same time inhibiting expansion of the other cell type (negative feedback) [19], [25], [26], [27]. To cite several examples, we note that the cytokine IL-6 is both necessary for Th17 differentiation and a known inhibitor of T_reg_ maturation [19], [20], [21], [28]. Similarly, mature Th17 cells secrete IL-17, which appears to have an inhibitory action on T_reg_ cells [29]. Likewise, the cytokine IL-2 seems to be necessary for the development of induced T_reg_ cells, while at the same time inhibiting Th17 cell differentiation [21], [24]. Finally, it appears that the transcription factors for both Th17 cells (ROR-γt, RORα) and T_reg_ cells (FOXP3) can bind to each other, thereby antagonizing one another even further and leading to proliferation of either one cell type or the other, but not both [17], [24], [30].

The interactions between Th17 cells and T_reg_ cells outlined above are captured using the following set of three equations.
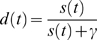
(1)

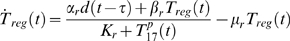
(2)

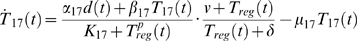
(3)


Equation (1) describes the relationship between the number of APCs actively engaged in antigen presentation, d(t), and the antigen load itself, s(t), where t is time. The parameter, γ, in equation (1) is thus the antigen load at which half of the available APCs are actively presenting antigen to T-cells. In equation (1) we make no distinction between the different types of APCs (eg. immature vs. mature dendritic cells [31]) that stimulate differentiation into the two T cell lines considered in the model(T_reg_ and Th17). Rather, we assume that a fixed amount of antigen will create a similar number of APCs primed for T_reg_ and Th17 cell differentiation.

Equations (2) and (3) describe the kinetics of the T_reg_ and Th17 cell populations respectively. In these equations T_reg_(t) and T_17_(t) are the sizes of the T_reg_ and Th17 cell populations, α_r_ and α_17_ are the rate constants governing T_reg_ and Th17 cell differentiation as a result of APC stimulation, β_r_ and β_17_ are the rate constants governing T_reg_ and Th17 cell differentiation as a result of positive feedback interactions, K_r_, K_17_ and p are parameters governing the strength and onset of negative feedback interactions, μ_reg_ and μ_17_ are the natural death rates of the two T cell types, δ is a parameter that determines how rapidly Th17 activation switches on as a function of T_reg_ produced TGF-β, and v is a term representing TGF-β production by all cell types other than T_reg_ cells. Since experimental observations suggest that T_reg_ cells produce the TGF-β necessary for Th17 cell development [20], [21], [22], we assume that v≪δ, meaning that the Th17 cell population does not expand in the absence of T_reg_ cells, at least for a properly functioning immune system.

Although equations (2) and (3) are similar in structure to a model that has been previously applied to the Th1/Th2 system [32], there are two crucial differences. The first relates to the nature of the input signal, d. In equation (2) we assume that the rate of T_reg_ differentiation at time t is determined by the level of APC stimulation that occurred τ time units earlier, d(t- τ), where τ is the additional amount of time it takes a naïve T cell to differentiate into a fully functioning T_reg_ cell compared to the time necessary for Th17 cell maturation. This modification is in keeping with the Abbas experiments on the lymphopenic mouse model [14], [33]. The second feature that is unique to our dynamics equations is the additional Hill function in equation (3). This term is included to reflect the constraint that Th17 cells cannot fully mature and/or survive without a low concentration of T_reg_ produced TGF-β. Both the time delay in equation (2) and the Hill function in equation (3) are characteristics that are unique to the T_reg_/Th17 system, and thus do not appear in the Th1/Th2 model. Interestingly, while both the time delay between T_reg_ and Th17 cell development and the pleiotropic role of TGF-β in immune defense have been noted experimentally, these aspects of immune system operation have remained difficult to rationalize according to current paradigms of immune action [34]. Both properties, however, are essential to GDP decision-making. Therefore, despite the mathematical simplicity of the GDP model, it effectively accounts for and explains some of the less intuitive mechanisms of immune regulation that have been witnessed empirically.

As will be shown in the remainder of the paper, the dynamic interactions between Th17 cells and T_reg_ cells enable the immune system to encode time-dependent information into relative T-cell population sizes. As these population sizes change through T-cell interactions with APCs and each other, the immune system comes to a final immunoregulatory decision by amassing either a large excess of T_reg_ cells, in which case the immune system develops peripheral tolerance towards the antigen, or else a large excess of Th17 cells, in which case a defensive immune response is initiated.

**Figure 1:**
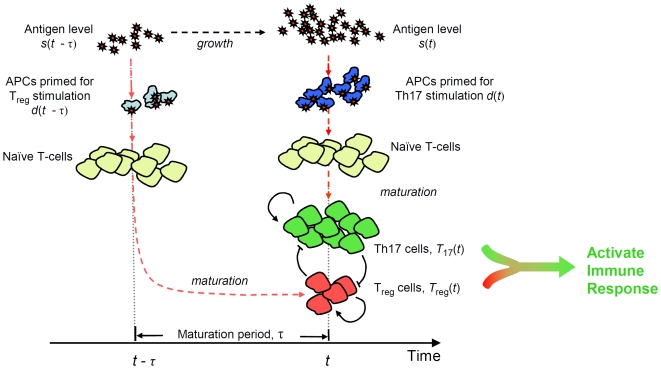
A schematic illustrating the key components of the Growth Detection Paradigm for immune system decision-making. In this case, the antigen is part of a growing population, and since this signals the threat of a pathogen invader, an immune response is initiated.

Before we move on to consider potential antigenic stimulation scenarios and their effects on the GDP decision-making process, we will first point out the general scope of the GDP model The purpose of this model is not to elucidate immune system operation in its entirety. Rather, GDP accounts for the fundamental ‘on/off’ decision that the immune system must make regarding its response to a particular antigen. As a result, we do not use the GDP model to consider interactions with additional types of effector cells, nor do we attempt to relate our predictions to the specific ‘variety’ of immune response that is mounted (eg. primarily Th1, primarily Th2, etc). Similarly, we do not discuss the steps leading from activation of an immune response to the development of memory cells primed for a subsequent encounter with the stimulating pathogen. Instead, we focus on whether or not regulatory T cells dominate, and thus whether or not peripheral tolerance is induced. Further discussion of the proposed model can be found in Supporting Information S1. A brief schematic of the interactions that we use in formulating the GDP model for the T_reg_/Th17 system is shown in Figure 1.

## Results

### GDP success and immune system regulation

In order to effectively discriminate between harmless antigenic substances and harmful pathogenic invaders, the GDP mechanism must (a) activate the immune system towards antigens from any growing population of pathogens, (b) induce peripheral tolerance of the immune system towards a constant stimulus from a foreign or self antigen, and (c) induce peripheral tolerance of the immune system towards a sudden injection of a foreign but non-replicating substance (e.g. venom from a wasp sting). Using the proposed dynamics model from equations (1) through (3), we show that the T_reg_/Th17 cell system satisfies these criteria for effective immune regulation. Moreover, we show that the T_reg_/Th17 cell system operates robustly even in the presence of a high level of noise.

**Figure 2:**
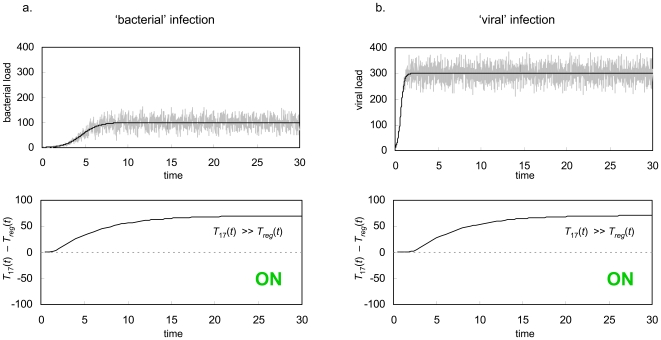
Logistic growth. GDP response (number of Th17 cells per unit volume minus number of T_reg_ cells per unit volume) to logistic growth (black line) perturbed by random noise (grey line). In (a) the pathogen has a slower growth rate (*r* = 1) and a lower carrying capacity (*K* = 100) while in (b) the pathogen has a faster growth rate (*r* = 5) and a higher carrying capacity (*K* = 300). For both scenarios an aggressive immune response is successfully initiated, as indicated by the relative abundance of Th17 cells compared to T_reg_ cells. The immune system parameters used for this simulation are *α_r_* = 15.1, *α*_17_ = 15.0, *β_r_* = *β*_17_ = 1.0, *μ_r_* = 1.0, *μ*_17_ = 1.1, *K_r_* = *K*_17_ = 1.1, *γ* = 1.0, *τ* = 1, *δ* = 1×10^−10^, *v* = 1×10^−14^, and *p* = 3.

In Figure 2, we consider the logistic growth of two different pathogens, one with a slow replication rate and a low carrying capacity (maximum pathogen load), and the other with a faster replication rate and a higher carrying capacity. For logistic growth of an antigen population, s(t), we have:
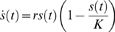
where *r* the pathogen growth rate, and K the pathogen carrying capacity. We term the slow replicating pathogen the ‘bacterial’ infection and the fast replicating pathogen the ‘viral’ infection. This is primarily to put into context the differing scales over which immune system activation can occur (see the Supporting Information S1), and the two simulations in Figure 2 could just as easily represent fast and slow replicating viruses, or fast and slow replicating bacteria. What is extraordinary is that, for wildly varying growth rates and pathogen loads, the same immune system parameters (in this case α_r_ = 15.1, α_17_ = 15.0, β_r_ = β_17_ = 1.0, μ_r_ = 1.0, μ_17_ = 1.1, K_r_ = K_17_ = 1.1, γ = 1.0, τ = 1, δ = 1×10^−10^, v = 1×10^−14^, p = 3. For further discussion of parameters, see Supporting Information S1) can accurately and robustly detect pathogen growth, ultimately triggering an immune response towards the hostile invader. In other words, growth detection by the T_reg_/Th17 system is highly robust, and results in an immune response to pathogen growth over a wide range of disease growth rates.

**Figure 3 pone-0008112-g003:**
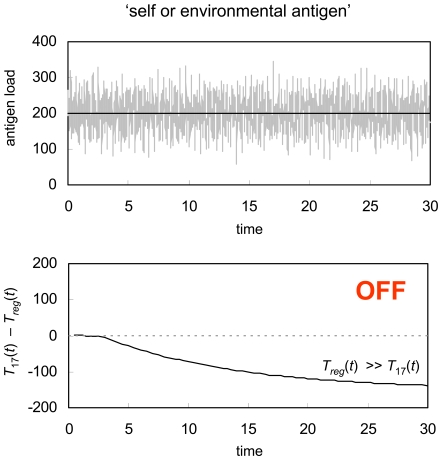
Constant stimulus. GDP response to a constant stimulus (black line) perturbed by random noise (grey line). In this case, T_reg_ cells significantly outnumber Th17 cells, thus the immune system develops peripheral tolerance to the signal, preventing a potentially deadly autoimmune disease or an allergic reaction. The immune system parameters used for this simulation are the same as in Figure 2.

In Figure 3, we show the opposite scenario – stimulation of the immune system by either a self or foreign antigen at a relatively constant level. In addition to the obvious example of host proteins, this scenario represents constant stimulation by commensal bacteria in the gut, or low level, chronic exposure to an environmental agent like pollen or dust mites. In this case, there is no extended period of antigen growth, and the same T_reg_/Th17 system as was used in Figure 2 clearly induces peripheral tolerance. Consequently, the host is protected against a destructive attack on its own tissue, or an excessive inflammatory reaction towards an antigen that poses no threat.

**Figure 4 pone-0008112-g004:**
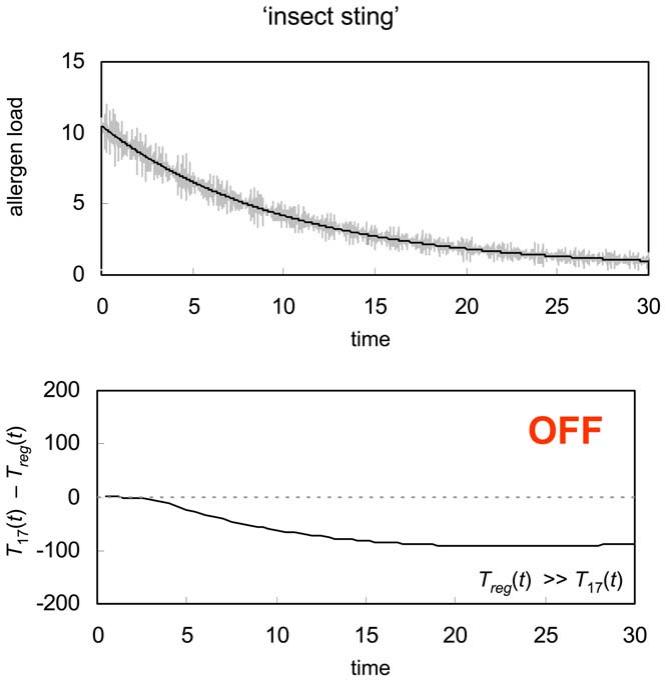
Injection. GDP response to a sudden injection of foreign material that subsequently decays exponentially(black line) perturbed by random noise (grey line). As in Figure 3, the immune system develops peripheral tolerance to the injected antigen, preventing a potentially deadly allergic reaction. The immune system parameters used for this simulation are the same as in Figures 2 and 3.

Not only is the immune system exposed to environmental antigens at steady-state levels but also, on occasion, the immune system can be subject to the sudden injection of a foreign substance which, after an initial rapid influx into the bloodstream will typically decay slowly until it eventually disappears from the host of its own accord. A wasp sting is a good example of this scenario. In Figure 4, we show how GDP responds to the sudden injection of a non-replicating stimulus. As with constant antigen exposure, this type of antigen time-profile leads to induction of peripheral tolerance, preventing the potentially deadly side effects of an unnecessary immune response, including excessive swelling, hives, nausea and even anaphylactic shock.

For the antigen stimulation scenarios outlined in Figures 2 – [Fig pone-0008112-g003] 4, we have shown how the T_reg_/Th17 system sets up a GDP mechanism for immune system regulation which can successfully discriminate between pathogens and innocuous self or environmental antigens, activating a defensive immune response in the first case, and inducing peripheral tolerance in the second. While GDP appears to be a robust decision-making mechanism in most circumstances, it will, now and then, fail, and this can lead to either chronic infection or an allergic reaction.

### GDP failure and immune system dysregulation

**Figure 5 pone-0008112-g005:**
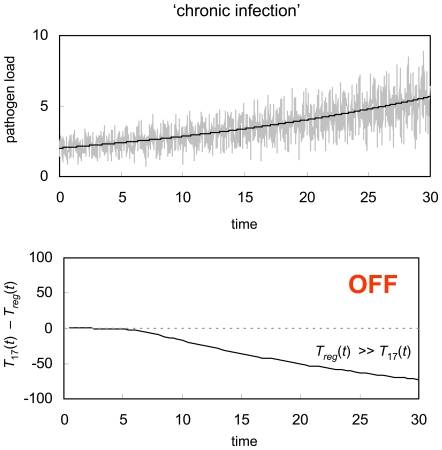
Chronic infection. GDP response to an extremely slow growing pathogen. As in Figure 2, this example assumes that the pathogen population undergoes logistic growth, however here the growth rate is much slower (*r* = 0.035), while the carrying capacity remains the same (*K* = 100). In this case, the immune system fails to mount a defensive response, developing tolerance to the antigen instead and thereby allowing for the establishment of a chronic infection. The immune system parameters used for this simulation are the same as were used in Figures 2 through [Fig pone-0008112-g003] 4.

Many chronic bacterial diseases, including tuberculosis and leprosy [35] are characterized by bacterium with extremely slow growth rates. At the same time, it has been suggested that the ability of these microorganisms to survive for long periods within the host may be due, in part, to the induction of peripheral tolerance as a result of a lacking ‘signal’ required for T cell activation [36]. We show that this as yet uncharacterized ‘signal’ is growth of the pathogen itself, and that slow growing pathogens may be particularly effective at inducing chronic infection because, among other immune evasion tactics that they employ, they can actively ‘trick’ the GDP mechanism. As an example, we consider leprosy. To emulate the 2 day doubling period of the leprosy bacterium relative to the more typical 100 minute doubling period of faster growing bacteria [37], we ran a simulation of the GDP system assuming a pathogen with a replication rate that is 29 times slower than the replication rate used to generate Figure 2 a). In Figure 5 we show that this extremely slow growing infection can, indeed, induce peripheral tolerance, minimizing T cell activation and potentially allowing the pathogen to invade the host against relatively little resistance.

**Figure 6:**
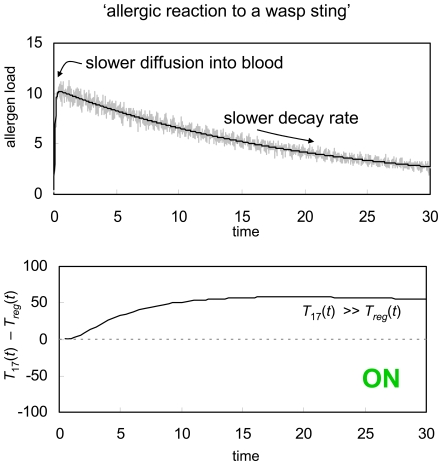
Allergic reaction. GDP response to a sudden injection of foreign material that subsequently decays exponentially (black line) perturbed by random noise (grey line). In contrast to Figure 4, the antigenic signal in Figure 6 both diffuses into the blood stream and decays more slowly. This causes the immune system to mount a defensive response, suggesting that the host will develop an allergy to the injected antigenic signal. The immune system parameters used for this simulation are the same as were used in Figures 2 through [Fig pone-0008112-g003] [Fig pone-0008112-g004] 5.

Just as the immune system occasionally fails to mount an adequate attack against a pathogen invader, it can also mistakenly trigger a vigorous immune response against a relatively harmless environmental antigen. When this happens, the stage is set for an allergic reaction, either immediately or, more commonly, upon a second encounter with that same stimulus. This type of immune system failure can also be explained in the context of GDP activation. As an example, in Figure 6 we repeat the GDP simulation for sudden injection of a non-replicating antigen (Figure 4). This time, however, we assume that the rate of diffusion of the antigen into and through the blood stream is slightly slower, and also that the foreign material has a slightly lower natural decay rate. In contrast to the earlier simulation, which suggested the development of peripheral tolerance towards the injection, the simulation in Figure 6 implies the onset of an immune response. In other words, this time the rapid influx of a small amount of foreign antigen either triggers an immediate allergic reaction, or else will lead to enough of an immune response that the immune system is primed and develops memory cells against the antigen, causing an allergic reaction upon any subsequent encounters with that same stimulus.

### Thymic T_reg_ cells

The GDP model explains the induction of peripheral tolerance and as a result focuses on induced T_reg_ cells that mature in the periphery. There is, however, a second population of regulatory T-cells that develops in the thymus [38]. These ‘natural T_reg_’ cells are generated in response to intrathymic self-antigens during the early stages of fetal and neonatal T-cell development [39], and are then exported to the peripheral tissue where they prevent activation of other self-reactive immune effector cells, thereby inhibiting autoreactivity. Natural T_reg_ cells form part of a system of immune regulation known as central tolerance. While central tolerance has been the primary paradigm of immune action for many years, it has long been recognized that central tolerance alone cannot explain immune decision-making in its entirety [20], [31], and that central tolerance must work in concert with peripheral tolerance in order to accomplish successful immune control.

**Figure 7:**
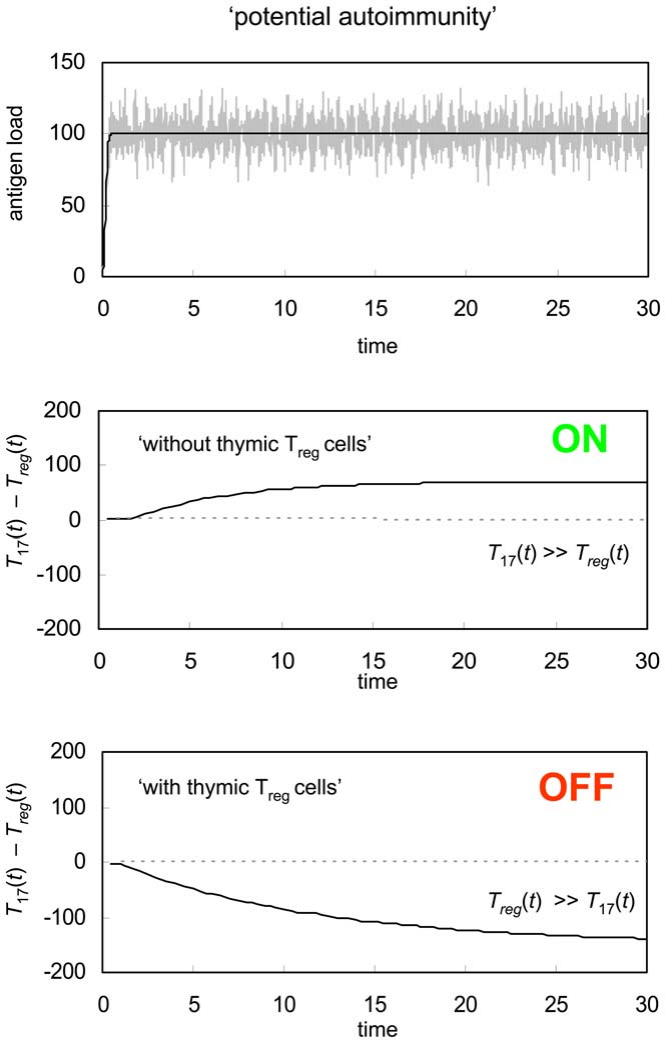
Protective effect of thymic T_reg_ cells. GDP response to an antigen with dynamics that trigger an immune response in the absence of any thymic T_reg_ cells. If a small population of T_reg_ cells (T_reg_(0) = 5) are present initially, however, tolerance is induced, thereby protecting the host from a potentially autoreactive immune response. The immune system parameters used for this simulation are the same as were used in Figures 2 through [Fig pone-0008112-g003] [Fig pone-0008112-g004] [Fig pone-0008112-g005] 6.

Although central tolerance and peripheral tolerance are, to some degree, separate immune regulation strategies, both can be effectively rationalized using the GDP mechanism. In order to show how natural T_reg_ cells and central tolerance can be incorporated into the basic GDP framework, we consider another scenario wherein the concentration of a self-antigen increases rapidly and then levels-off. Figure 7 shows how, in the absence of any natural or induced T_reg_ cells with specificity towards the self-antigen (T_reg_(0) = 0) the GDP model predicts immune activation and autoimmunity. When, however, there is a small, pre-existing population of natural T_reg_ cells (T_reg_(0) = 5), the same antigenic signal gives rise to tolerance. In other words, relatively low levels of natural regulatory T-cells can provide protection such that the immune response becomes refractive to time-dependent changes in an antigenic signal that would otherwise drive the immune system towards activation.

### Model parameters

**Figure 8:**
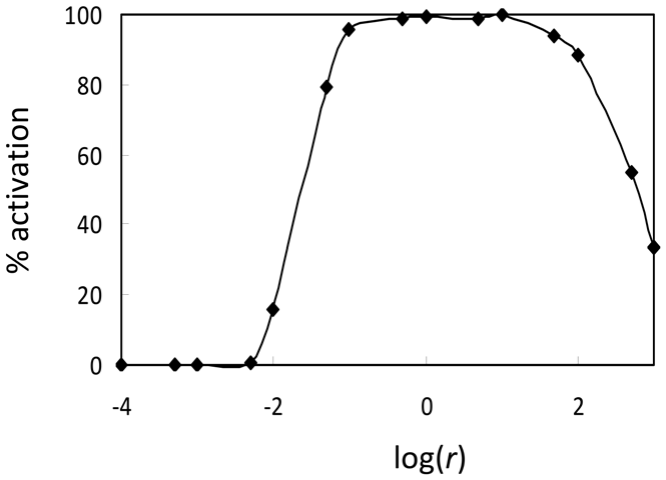
Percentage of randomly selected immune parameter sets that activate as a function of pathogen growth rate, *r*. For this figure we use *α_r_* ∈ [1, 100], 0.99*α_r_*<*α*_17_<*α_r_*, 0.1*α_r_*<*β_r_*<0.5*α_r_*, 0.99*β_r_*<*β*_17_<*β_r_*, *β_r_*/*K_r_*<*μ_r_*<1.05*β_r_*/*K_r_*, *μ_r_*<*μ*_17_<1.01*μ_r_*, *K_r_* ∈[1], [10], *K_r_*<*K*_17_<1.01*K_r_*, *γ *∈[1], [10], 30*α_r_* (*β_17_*/*K_17_*−*μ_17_*+*β_r_*/*K_r_*−*μ_r_*)/*K_r_*<*τ*<100*α_r_* (*β_17_*/*K_17_*−*μ_17_*+*β_r_*/*K_r_*−*μ_r_*)/*K_r_*, *δ* = 1×10^−10^, *v* = 1×10^−14^, and *p* = 3. Parameter ranges and justifications for parameter relationships are discussed further in Supporting Information S1.

All of the simulations in Figures 2–6 use the same set of model parameters. Fortunately, however, successful GDP decision-making does not rely on a specific set of carefully tuned kinetic rate constants. Rather, GDP can be generated over a wide range of parameter values, provided certain parameter relationships are maintained. In Figure 8, for instance, we show the percentage of randomly selected GDP parameter sets that result in successful immune system activation as a function of pathogen growth rate. Notice that nearly 100% of the randomly selected parameter sets lead to activation for pathogen growth rates between r = 0.1 and r = 50, while almost no parameter sets cause activation for growth rates below r = 0.005 (i.e. none of the parameter sets would cause immune activation in response to a steady-state or decreasing antigenic signal).

Although GDP can be generated by a wide range of different parameter values, certain parameter relationships are required for successful immune defense and, if these parameter relationships are varied, the immune system can become more or less sensitive to different antigen stimulation scenarios. Broadly speaking, for example, decreasing α_r_ and τ relative to μ_r_ and μ_17_ or increasing μ_r_ and μ_17_ relative to β_r_ and β_17_ will tend to reduce the risk of allergic reaction (for a complete discussion of parameter relationships, see Supporting Information S1). While reducing the risk of allergic reaction might seem optimal for successful immune operation, further analysis shows that the same changes which decrease allergic tendencies will also lower the sensitivity of the immune system to slow-growing pathogens, leading to a higher risk of chronic infection. This apparent trade-off between the risk of allergic reaction and the risk of chronic infection is one of the less intuitive results of our model and bears an intriguing resemblance to the ‘hygiene hypothesis’ [40], [41]. According to the ‘hygiene hypothesis’ the prevalence of allergies in the westernized world is a result of limited exposure to infectious diseases during early development. Unfortunately, this theory has remained somewhat controversial [42] in part because a mechanistic link between allergies and chronic infection (e.g. by helminth worms) has remained elusive. The GDP paradigm, however, offers a potential explanation for this relationship by highlighting the trade-off associated with defense against slow-growing pathogens and protection against allergic overreaction.

In addition to suggesting a mechanism for the hygiene hypothesis, the sensitivity/robustness trade-off that we observe during GDP operation means that there is no perfect set of parameters that can allow for optimal immune system behavior under all antigen stimulation scenarios. It is therefore not surprising that different people arrive at different solutions with respect to the balance between pathogen defense and propensity towards allergic reaction. Furthermore, based on observations including the hygiene hypothesis, we suggest that while certain immune system parameters might be set by genetics, others are likely tuned during early childhood in response to the degree and/or type of antigenic stimulation typically encountered. This tuning during childhood would merely involve slight adjustments to the rates of stimulation, proliferation and turnover of the T_reg_ and Th17 cell populations, and would allow for context specific optimization of the tolerance/activation thresholds such that a person's immune system was tailored to the specific range of pathogen growth rates that they might typically encounter during their lifetime.

## Discussion

Despite the importance of immune system regulation with respect to pathogen defense, allergic reactions and autoimmunity, an explanation for the generation and maintenance of immune system activation and peripheral tolerance has remained elusive. Most explanations for immune system regulation fall into what we will term the ‘missing signals’ category – these theories suggest that the immune system is triggered to respond aggressively when it receives several simultaneous signals indicating the presence of a pathogen, but induces tolerance when certain of these multiple signals are absent. While GDP is, essentially, a ‘missing signals’ model as well, in GDP the missing signal is not a chemical factor (eg. cytokine) or spatial pattern (eg. epitope, TLR activation, etc.), but rather, the temporal pattern of growth itself.

The GDP model bears some similarity to the model proposed by Steinman and Nussenzweig [31], who suggested that continued steady state stimulation of T cells by self-antigen presenting immature dendritic cells (iDC) might tip the immune response towards tolerance. While the steady-state stimulation hypothesis can explain why the immune system does not react towards all antigens, it does not provide a complete explanation for the opposing process of immune activation, particularly as it applies to aggressive immune responses that occur in the absence of any discernable TLR stimulation or other pattern recognition mechanisms. A complete theory of peripheral tolerance, however, must account for both activation and suppression signals and must additionally delineate how these two opposing signals are integrated for the eventual task of discriminating between dangerous pathogens and innocuous self and environmental materials.

The GDP mechanism differs from the Steinman and Nussenzweig hypothesis because GDP suggests that it is not steady-state stimulation in and of itself that gives rise to immunologic tolerance, but instead, it is the absence of growth. As a result, GDP can rationalize both immune system activation and immune system suppression independent of any other signaling pathways. In addition, the GDP mechanism predicts a slightly different immune response to a steady state antigenic signal. More specifically, because of the positive and negative feedback mechanisms involved in the interplay between T_reg_ and Th17 cells, the GDP system exhibits hysteresis. Therefore, GDP predicts that the immune system may or may not react to a seemingly steady-state antigenic signal depending on whether or not the antigen exhibited a brief period of growth when it first appeared in the host. This is in direct opposition to the prediction made by the Steinman and Nussenzweig hypothesis, and will almost certainly prove crucial in rationalizing immune system activation against certain self antigens and environmental agents.

In this paper, we have introduced what we term the ‘Growth Detection Paradigm’ (GDP) for immune system activation. Using a simple model that is based on the T_reg_/Th17 system, we have shown how GDP emerges naturally as a result of the signaling interactions and maturation kinetics of Th17 and T_reg_ cells. We have also shown that GDP is a particularly effective means of discriminating between innocuous antigens and pathogenic invaders. Most notably, GDP can detect the presence of pathogens with a nearly 100% accuracy over an almost 3 orders of magnitude range of pathogen growth rates. In addition, GDP is amazingly robust against high frequency noise (see, for example, Figures 2–6). In addition, we have shown that efficient GDP detection is not dependent on a narrow region of finely tuned parameters, but rather, can be obtained for a wide range of different parameter values, provided specific parameter relationships are maintained (for further discussion of the parameter values, see Supporting Information S1). Finally, we have outlined the implications that a GDP interpretation has on everything from chronic infection to allergies, and we have shown how GDP can rationalize the observed responses of the immune system to various types of pathogenic, environmental and self antigens.

The GDP model by no means lessens the importance of other, more traditional views of immune system activation. In fact, we do not expect GDP to replace immune system activation schemes involving spatial pattern recognition (eg. TLRs, TCRs, BCRs, etc.). Rather, we view GDP as working in parallel with these other mechanisms. For example, strong TLR stimulation will almost certainly circumvent the GDP pathway entirely, while GDP induced T_reg_ up-regulation might quell an immune response initiated by weak TLR engagement. To that end, we note that the easiest system in which to study GDP is a setup similar to the Abbas lymphopenic mouse model, since this system eliminates many of the other mechanisms of immune regulation (eg. TLR activation) that may overshadow GDP itself.

While we have focused on the interplay between T_reg_ cells and Th17 cells, the immune system's ability to monitor growth is likely far more complex and sophisticated than the simple model that we present here. Time-dependent information, for instance, will almost certainly prove to be encoded not only in the relative sizes of the T_reg_ and Th17 cell populations, but also in the population sizes of other cell types, including Th1 and Th2 helper T cells, and possibly even APCs like dendritic cells and macrophages. Therefore, the ‘activation versus suppression’ step that we have outlined in this paper is meant to be taken as a significant approximation to the real system, which likely involves further decision-making processes made either in parallel with, or else following after the T_reg_/Th17 decision itself. As a result, although the insight from our simple GDP model can be used to interpret some of the mysteries surrounding immune system activation, pathogen defense and allergies, we expect that even greater strides towards understanding the immune system will be made once we extend the GDP model to consider other immune effector cells.

In spite of the increase in complexity that additional cell populations will bring to the GDP model, at its heart, GDP bears one of the hallmarks that we have come to expect of biological problem-solving systems – it is remarkably simple. GDP relies on different cell populations with different maturation rates encoding information from different time points, which then allows the immune system to determine whether or not a particular antigenic signal is growing. Once growth, or the lack thereof, has been detected, positive and negative feedback loops between the different T cell populations force the immune system into one of two possible steady states causing a switch-like ‘on/off’ response that stabilizes the decision to either develop peripheral tolerance towards the antigen, or else respond through active defense. We anticipate that this simple, yet elegant ‘Growth Detection Paradigm’ will prove to be pervasive throughout different elements of the vertebrate immune system, and that it may turn out to be the proverbial ‘missing piece of the puzzle’ that immunologists have been searching for in their quest to understand immune system regulation. As a result, we expect that GDP will figure prominently in both developing an understanding of fundamental immune system behavior and controlling pathological consequences associated with immune system dysregulation, including chronic disease, vaccination failure, allergies, autoimmunity and even organ rejection during transplants.

## Supporting Information

Supporting Information S1 Further mathematical analysis of GDP model.
(0.42 MB DOC)
