# Supplementary material for: The Danger Is Growing! A New Paradigm for Immune System Activation and Peripheral Tolerance
Source: PLoS One. 2009 Dec 2;4(12):e8112. doi: 10.1371/journal.pone.0008112 (PMC2779512; doi:10.1371/journal.pone.0008112)
Supplement: Supporting Information S1 — Further mathematical analysis of GDP model. (0.42 MB DOC) [file pone.0008112.s001.doc]

Supporting Information

## Model Analysis. In what follows, we outline the basic mathematical underpinnings of the GDP model, focusing on the dynamic behavior of the Th17 and T_reg_ cell populations during the immune decision-making process. This analysis gives a more in depth understanding of how GDP is generated by the T_reg_/Th17 system and also shows how relationships between kinetic parameters can effect immune operation which, in turn, allows us to point to further empirical experimentation that should be done to better characterize immune regulation by the GDP mechanism. To reiterate, the equations used to model the T_reg_/Th17 system are as follows.

$d(t)=\frac{s(t)}{s(t)+\gamma}$ (1)

(2)

(3)

where all parameters and terms are as described in the main text.

## A. The Initial Phase. The initial phase of the immune response can effectively set the stage for the entire immune decision-making process. As a result, we begin our analysis by focusing on how GDP operates during the early stages of antigen encounter by a naïve immune system. Since we are primarily concerned with determining which of the two T-cell varieties dominates and thus whether tolerance or activation is invoked, we will specifically consider the evolution of the size difference between the two T-cell populations, Δ = *T*_17_ - *T_reg_*. From equations (2) and (3) we can describe the dynamics for the evolution of Δ as

(4)

To simplify equation (4), we begin by considering an APC signal that is approximately linear in time, such that $d(t-\tau)\approx d(t)-k\tau=d-k\tau$. This simplification makes it easier to analyze equation (4) in terms of increasing (*k* > 0), decreasing (*k* < 0) and constant (*k* = 0) antigenic signals. Equation (4) can be simplified further by noting that when an immune system is naïve to a stimulating antigenic signal it has neither mature T_reg_ nor mature Th17 cells with specificity towards that antigen. As a result, during the first phase of the immune response, both T_reg_ and Th17 cell populations will be relatively low, such that *T_reg_*(*t*) << *K*_17_ and *T*_17_(*t*) << *K_r_*. When these inequalities hold and when, additionally, the mature T_reg_ population has had sufficient time to produce at least a few mature effector cells ( *T_reg_* >> *δ*, *T_reg_* >> *v* ), we can simplify equation (4) to

(5)

In equation (5) we have suppressed time for notational simplicity, although we note that Δ, *d,* *T_reg_* and *T*_17_ are all functions of time.

According to the GDP regulation mechanism, the immune system shifts towards activation when and towards peripheral tolerance when . In what follows, we use these criteria in order to establish relationships between relative parameter values and immune system behavior. In particular, we focus on the overall activation tendency of the immune system and on its robustness to noise.

*Tolerance vs. Activation*: In a properly functioning immune system, tolerance should be induced for both decreasing (*k* < 0) and constant (*k* = 0) antigenic signals, while activation should be induced for increasing (*k* > 0) antigenic signals. Notice that these conditions for tolerance and activation are asymmetric to the extent that a constant antigenic signal (*k* = 0) must lie firmly within the basin of attraction of the tolerant state. If, instead, the *k* = 0 signal was positioned directly on the basin boundary effective GDP would be impossible, as the immune response to a constant antigen would then depend entirely on noise, and this would result in high instances of autoimmunity and chronic allergies.

In order to determine the parameter relationships necessary to ensure robust immune response to a constant antigenic signal, we set *k*  = 0 and consider a scenario in which the immune system is perfectly naïve (Δ = 0). This leaves only the second and fourth terms in equation (5), and from these we see that the conditions $\alpha_{r}/K_{r}>\alpha_{17}/K_{17}$, $\beta_{r}/K_{r}>\beta_{17}/K_{17}$ and $\mu_{r}<\mu_{17}$ will ensure GDP evolution towards peripheral tolerance in the context of a constant antigen load.

While the above inequalities must hold in order to establish tolerance towards constant stimulation by self and environmental antigens, when the inequalities are too large, the immune system will tend to be excessively tolerant and, as a result, it will disregard not only constant antigens but also to slow-growing pathogens. In other words, the ability of bacteria to evade immune detection by reducing replication rate depends crucially on the exact magnitudes of the above inequalities. Therefore, while we expect that healthy immune systems will be characterized by most, if not all of the above inequalities, we also suggest that these inequalities should be relatively small, so as not to open up a large window of opportunity for pathogen circumvention of GDP.

Phase diagrams showing regions of activation and tolerance can be used to illustrate the overall activation tendency of the immune system as a function of the inequalities discussed above. In Figure 1 of the Supporting Information, for example, we consider the minimum detectable pathogen growth rate, *r*, in terms of the ratio $\alpha_{r}/K_{r}:\alpha_{17}/K_{17}$. As suggested, increasing $\alpha_{r}/K_{r}$ relative to $\alpha_{17}/K_{17}$ tips the balance in favor of peripheral tolerance, and this effect is clearly visible in the figure, where a larger region of tolerance appears at higher values of $\alpha_{r}/K_{r}:\alpha_{17}/K_{17}$. The parameters used to generate Figure 1 are as follows: *α*_17_ = 15.0, *β_r_* = *β*_17_ = 1, *μ_r_* = *μ*_17_ = 1.0, *K_r_* = *K*_17_ = 1.1, *γ* =1.0, *τ* = 1, *δ* = 1 × 10^-10^, *p* = 3, *κ* =1000, *v* = 1 × 10^-14^ and variable *α_r_* (over a range from *α_r_* = 15.01 to *α_r_* = 22.5) The phase diagram was derived from the full model in equations (1) through (3).

In Figure 2, we similarly consider the minimum detectable pathogen growth rate as a function of the ratio $\beta_{r}/K_{r}:\beta_{17}/K_{17}$. Again, increasing $\beta_{r}/K_{r}$ relative to $\beta_{17}/K_{17}$leads to a larger region of tolerance, and this is in keeping with the analysis above. The parameters used to generate Figure 2 are as follows: *α_r_* = *α*_17_ = 15.0, *β*_17_ = 1, *μ_r_* = *μ*_17_ = 1.0, *K_r_* = *K*_17_ = 1.1, *γ* =1.0, *τ* = 1, *δ* = 1 × 10^-10^, *p* = 3, *κ* =1000, *v* = 1 × 10^-14^ and variable *β_r_* (over a range from *β_r_* = 1.01 to *β_r_* = 3). Again, the full model was used.

Finally, in Figure 3, we consider the minimum detectable pathogen growth rate as a function of the ratio $\mu_{r}:\mu_{17}$. In this case, increasing $\mu_{r}$ relative to $\mu_{17}$ decreases the region of tolerance, as expected based on the analysis above. The parameter set used to generate Figure 3 is as follows: *α*_17_ = *α_r_* = 15.0, *β_r_*. = *β*_17_ = 1, *μ*_17_ = 1.0, *K_r_* = *K*_17_ = 1.1, *γ* =1.0, *τ* = 1, *δ* = 1 × 10^-10^, *p* = 3, *κ* =1000, *v* = 1 × 10^-14^ and variable *μ_r_* (over a range from *μ_r_* = 0.001 to *μ_r_* = 1). Again, the full model was used.

Although the preceding arguments regarding the parameter ratios *α_r_/K_r_:α_17_/K_17_*, *β_r_/K_r_:β_17_/K_17_* and *μ_r_:μ_17_* should hold for healthy immune systems, there is no reason to believe that they will also hold for immune systems that are either autoreactive, or immunodeficient. In particular, a possible route to autoimmunity would be a significant reversal in one or more of the above inequalities, while immunodeficiency could be induced by increasing the same parameter ratios.

*Robustness to Noise*: In addition to favoring a regulatory response, effective GDP decision-making must be robust against noise and short time-scale efforts of the pathogen to circumvent the GDP detection mechanism. This means that the immune system must have the ability to reverse its initial activation or tolerance decision provided it receives a counter signal within a reasonable period of time.

The relative contributions of the immune parameters towards GDP robustness can again be understood by considering equation (5). In particular we focus on the first and third terms of equation (5), since we have already argued that the net effect of the second and fourth terms should be relatively small. In general, the first term in equation (5) is positive for an increasing (*k* > 0) antigenic signal, and negative for a decreasing (*k* < 0) antigenic signal, meaning that this term will tend to push the immune system towards activation if there is instantaneous growth in the antigen load and towards tolerance if there is instantaneous decay. In each case, the absolute rate of evolution towards activation or tolerance will depend on the magnitude of the rate of change of the antigen load as well as on the parameters *α_r_*, *K_r_* and *τ*. In particular, large values of *α_r_* and *τ*, and small values of *K_r_* will tend to cause the immune decision to evolve more rapidly by enhancing the rate of immune activation when the antigenic signal is increasing (*k* > 0) and simultaneously enhancing the rate of immune suppression when the antigenic signal is decreasing (*k* < 0). By accelerating the rate of evolution towards a decision, these parameter values will also tend to make the immune system both more sensitive to slow growing pathogens and less resilient to noise.

As was done above, we consider the relationships between the parameters *τ,* *α_r_* and *K_r_* and immune system behavior in terms of phase diagrams (as before, based on the full model). In Figure 4a. we show the effect of increasing *τ* on immune system sensitivity to slow growing pathogens (low *r*), while in Figure 4b. we show the effect of increasing *τ* on robustness to noise (high *r*). As suggested by the analysis above, higher values of *τ* lead to larger regions of activation (higher sensitivity) at low antigen growth rates. On the other hand, higher values of *τ* are also associated with larger regions of activation at high antigen growth rates (lower robustness), which may cause the immune system to react to short time-scale changes in antigen concentrations like those associated with insect stings, injections and noise.

In Figures 5a. and 5b. we again consider immune responsiveness, this time as a function of *α_r_* (where, in contrast to Figure 1, we simultaneously change both *α_r_* and *α_17_*) As was the case in Figure 4, in Figure 5 it is clear that higher values of *α_r_* increase the region of activation at both the low and high rates of change in the antigen load. Therefore, like an increase in *τ*, an increase in *α_r_* is simultaneously associated with increased sensitivity towards slow growing pathogens, and decreased robustness against noise. This is in keeping with the discussion above.

Unlike the first term in equation (5), the third term does not depend on the instantaneous growth or decay rate of the antigenic signal, but rather, acts in response to the current state of the immune system itself. More specifically, this term will tend to reinforce the immune decision already underway when parameters are such that *Ω_17_* + *Ω_r_* > 0 and will tend to resist the existing immune decision when parameters are such that *Ω_17_* + *Ω_r_* < 0. . Intuitively, the condition *Ω_17_* + *Ω_r_* < 0 gives rise to decision reversibility (and thus robustness) because high T-cell death rates can effectively erase the initial immune response, providing a ‘virtual spring’ that acts against decision in either direction (i.e. it reverses both activation and tolerance decisions, returning the system to its naïve state). In terms of immune action, this robustness is crucial, since it prevents the immune system from reacting to antigen dynamics that are too fast to be associated with pathogen growth, and thus are more likely to be attributed to noise and/or antigen diffusion within the blood (ie during an injection or wasp sting). Based on this line of reasoning, we suggest that properly functioning immune systems should be characterized by cell replication and death rates such that *Ω_17_* + *Ω_r_* < 0.

While the ability of the immune system to reverse its decision during the early phase of the immune decision-making process is crucial for GDP robustness in a noisy environment, when *Ω_17_* + *Ω_r_* << 0, the immune system runs the risk of ignoring pathogens that replicate rapidly and reach carrying capacity on a short time scale. In Figures 6a. and 6b. we again consider phase diagrams, this time as a function of the quantity *Ω_17_* + *Ω_r_*. Once again, the trade-off between sensitivity and robustness is strikingly apparent, with higher (more positive) values of *Ω_17_* + *Ω_r_* giving rise to increased regions of activation at both high and low rates of change in the antigen concentration.

**B. The Intermediate Phase.** While the early phase of the immune response largely governs the ultimate decision, the intermediate and final phases solidify this decision and stabilize it such that, beyond a critical point, reversibility of the immune decision is no longer possible. This aspect of the GDP mechanism is necessary to ensure that neither saturation of APCs nor the arrival at the pathogen carrying capacity can reverse immune activation. In this section, we consider the evolution of the GDP response through the intermediate phase of the decision-making process.

As the immune response develops, the populations of both mature T_reg_ and mature Th17 cells expand in response to stimulation by APCs bearing the antigen. During this phase of the immune response, there is an increase in negative feedback interactions between T_reg_ and Th17 cells and, consequently, the nonlinearities in equations (2) and (3) become significant. As a result of these nonlinearities, equation (4) can exhibit bistability. In other words, for the same rate of change in the antigen population, the difference between the two T-cell population sizes, Δ, will often have two stable steady states. One way to illustrate this property of the GDP system is to treat the APC stimulation signals, *d*(*t*) and *d*(*t* – τ), as parameters and then to consider the dynamical properties of equation (4) in terms of these parameters. In essence, this amounts to assuming a separation of timescales such that the T_reg_ and Th17 cell populations equilibrate much more rapidly than the timescale associated with changes in the APC cell population. While this is not necessarily true for real immune systems, the insight that can be gained from this type of interpretation is, nevertheless, illustrative in terms of understanding the mechanisms of GDP action. Figure 7, for example, shows the steady states of equation (4) as a function of *d*(*t*), at the fixed value *d*(*t* – τ) = 0.015.

From Figure 7, it is clear that a strongly decreasing antigenic signal will generate a single steady-state with Δ < 0 (immune suppression) and a strongly increasing antigenic signal will generate a single steady-state with Δ > 0 (immune activation). However, for a range of antigen-time profiles, including constant antigenic stimulation, *d*(*t*) = *d*(*t* – τ), both activated and suppressed steady-states are possible. This bistability is important in terms of immune-regulation for several reasons. First, the bistability stabilizes the immune decision against noise, such that an immune system tending towards activation will require a *significant* *decrease* in the antigen population to revert to the suppressed state while an immune system tending towards suppression will, similarly, require a *significant* *increase* in the antigen population to revert to the activated state. Second, the bistability allows the GDP mechanism to operate effectively against pathogen populations that exhibit logistic growth up to a carrying capacity. In this case, even though the antigen population ceases to increase once the pathogen has reached its carrying capacity, the immune system will remain in the activated steady-state as a result of the bistability and associated phenomenon of hysteresis in the GDP system. Notice, in Figure 7, that a constant signal can leave the immune system in *either* the activated or suppressed state, depending on whether or not it follows a phase of antigen growth. More specifically, if the immune system begins in an activated state, a constant signal will result in an activated immune response. If, however, the immune system begins in the naïve or suppressed state, the constant signal falls into the basin of attraction for the suppressed state. This is a necessary condition for reliable GDP operation, since all antigenic signals will eventually reach a constant level (either because the pathogen itself reaches a carrying capacity, or else because of the inherent limited supply of APCs).

In Figure 7, *d*(*t – τ*) is fixed at a constant value. During engagement of the immune system, however, both the time-delayed APC signal, *d*(*t – τ*), and the real-time APC signal, *d*(*t*) change in response to the dynamics of the antigen population itself. As a result, an improved understanding of the GDP mechanism comes from considering the steady states of equation (4) as a function of both *d*(*t – τ*) and *d*(*t*). Figure 8 shows 3-D surfaces of these steady states for small values of *d*(*t – τ*) and *d*(*t*).

Figure 8 is useful to illustrate evolution of the immune decision-making process under the GDP mechanism. More specifically, as the antigen population changes with time, it causes an associated increase and/or decrease in the APC cell population through equation (2). The response of the APC cell population to a particular antigenic signal can, therefore, be represented by a specific trajectory on the *d*(*t*) *– d*(*t – τ*) plane. Furthermore, if T_reg_ and Th17 cell populations equilibrate rapidly compared to the timescale associated with APC cell dynamics, the approximate evolution of the decision criteria, Δ, can be visualized as the trajectory on the steady-state surfaces in Figure 8 that corresponds to the APC cell trajectory defined on the *d*(*t*) *– d*(*t – τ*) plane. Again, the assumptions made to arrive at this interpretation are not necessarily valid in real immune systems, but the analysis is nonetheless illustrative in terms of developing an understanding of GDP operation.

What is immediately obvious from the surface trajectory interpretation is the significance of the cusp catastrophe surface that appears at higher values of *d*(*t* – *τ*) in Figure 8. In particular, it is clear that for very low APC/antigen concentrations, the decision reached by the immune system regarding the threat level of the antigenic stimulus can be reversed easily, since there is no bistability. Once, however, APC/antigen concentrations have reached a critical level, bistability appears, and the associated hysteresis makes reversal of the immune decision much more difficult. If, for example, the immune system is in the activated state marked by the ‘×’ in Figure 8, the APC cell population, *d*(*t*), must decrease *significantly* in order to push the state of the system over the edge of the activated surface. When this occurs, the immune system will drop discontinuously from the activated state to the suppressed state, and will remain in the suppressed state unless the APC cell population exhibits a significant increase that can take the system back up to the activated surface. The projection of the fold surface in Figure 8 onto the *d*(*t*) - *d*(*t – τ*) plane gives the stability diagram shown in Figure 9.

Figure 10 shows a similar diagram, however this time we consider the factor increase in the APC signal that must occur over the time period τ in order to tip the immune system into either activation or peripheral tolerance. In addition, we divide the bistable region according to how a naïve immune system would evolve. Notice, in Figure 10, that higher antigen population growth rates are necessary to stimulate activation at low APC levels. Consider, for example, *d*(*t* - τ) < 0.001. At this APC level, the APC signal must more than double in order to stimulate an activated immune response. In contrast, when *d*(*t* - τ) > 0.04, activation merely requires an APC increase of approximately 25% over the same time period. Likewise, reversal of the activation decision also requires a more significant antigen decrease at higher APC levels. At *d*(*t* - τ) = 0.001, for instance, the immune system will revert to tolerance unless the antigenic load continues to double over τ. At *d*(*t* - τ) = 0.04, however, the antigen load must actually decrease over τ in order to reverse the direction of immune system evolution. In other words, at low antigen loads (and thus low APC levels), the immune system exhibits a high degree of plasticity with respect to the immune decision. This results in an immune system that is robust against noise during the initial stages of infection when noise is likely high due to the low pathogen load and the associated effects of stochasticity in small populations. At high antigen loads (and thus high APC levels), however, the immune decision is much harder to reverse. Not only does this transition from a reversible response to an irreversible response have relevance to the smooth operation of the GDP mechanism, but also, it has implications with respect to the dependence of immune activation on absolute antigen loads. In particular, although the size of the antigen load does not *per se* dictate whether or not the immune system reaches an activation or tolerance decision, it does affect the immune system’s tendency to reverse the initial immune decision. If, for instance, an immune response is mistakenly activated at low antigen loads, the immune system will naturally correct itself as soon as the antigenic signal growth rate reverses (or even, at very low antigen loads, slows down sufficiently). On the other hand, if an immune response is mistakenly activated at high antigen loads (eg. an organ transplant), it will *not* tend to revert to a tolerogenic state. This property of the GDP system could, particularly based on experimental evidence, be attributed to a threshold phenomenon. We suggest, however, that threshold models negate the higher level of sophistication implicit in the GDP regulation mechanism.

**C. The Final Decision.** Regardless of the nature of the antigen, the APC cells will, eventually, produce a nearly constant signal, either because the antigen signal itself has become constant (eg. the pathogen has reached its carrying capacity) or else because the antigen load has fully saturated the APC cell population. At this point in the immune response, the immune system will have reached a decision regarding the threat level associated with the antigen in question. While this is not the end of the immune response itself, it is the end of the GDP decision-making process and, depending on whether activation or tolerance has been selected, additional immune cells can develop to provide effector functions like pathogen clearance and memory.

In general, if the immune system has experienced sufficient antigenic stimulation during the decision-making phase of the immune response, there will be either a large excess of T_reg_ cells and hardly any Th17 cells (suppressed state), or else a large excess of Th17 cells and hardly any T_reg_ cells (activated state). Under these conditions, it is usually possible to simplify equations (2) and (3), depending on whether the suppressed state or the activated state has been reached. This allows us to find approximate expressions for Δ at the end of the GDP decision-making process.

Suppressed State: In the suppressed state, there will be a large excess of T_reg_ cells, and hardly any Th17 cells. Though exceptions can be found, in most cases, the large excess of T_reg_ cells and the limited number of Th17 cells means that the suppressed state will be characterized by T-cell population with *T_reg_* >> *K*_17_ and *T*_17_ << *K_r_*. As a result, equations (2) and (3) can be simplified to

(6)

(7)

where *d** is the final (constant) APC signal, with *d** ≈ 1 when the APC population is saturated, and *d** < 1 otherwise. In equation (7) we have additionally assumed that *T_reg_* >> *δ* and *T_reg_* >> *v*, since *δ* is expected to be significantly less than *K*_17_, and *v* is expected to be less than δ. Solving for the steady-states of equations (6) and (7), *T_reg_** and *T*_17_*, and substituting these into the expression for the decision criteria, Δ, we find

$\Delta*=T_{17}^{*}-T_{reg}^{*}\approx\frac{\alpha_{17}d^{*}}{\mu_{17}x^{q}-\beta_{17}}-x, x=\frac{\alpha_{r}d^{*}}{\mu_{r}K_{r}-\beta_{r}}$ (8)

Activated state: In the activated state, there will be a large excess of Th17 cells, and hardly any T_reg_ cells. Similar to the scenario for the suppressed state, then, this will typically mean that equations (2) and (3) can be simplified based on the relationships *T*_17_ >> *K_r_* and *T*_reg_ << *K*_17_. This gives

(9)

(10)

In equation (10) we have once again assumed that *T_reg_* >> *δ*. This will, in general, hold for real immune systems, since a very low concentration of TGF-*β* is sufficient to stimulate Th17 production, and thus even when T_reg_ levels have been suppressed, there should still be sufficient TGF-*β* to permit Th17 maturation. Solving for the steady-states of equations (9) and (10) and substituting these into the expression for the decision criteria we find

$\begin{matrix} \Delta*=T_{17}^{*}-T_{reg}^{*}\approx-\frac{\alpha_{r}d^{*}}{\mu_{r}y^{p}-\beta_{r}}+y \\ y=\frac{\alpha_{17}d^{*}}{\mu_{17}K_{17}-\beta_{17}} \end{matrix}$ (11)

where *d*^*^ is, as before, the APC level at the end of the decision phase. Equations (8) and (11) relate the eventual difference between T_reg_ and Th17 cells to the kinetic parameters for immune activation, and thus may, in the future, be useful for characterization of GDP parameters from experimental systems.

**D. Parameters.** As was suggested in the main text, kinetic parameters for T_reg_ and Th17 cell proliferation, expansion and turnover almost certainly exhibit wide variation between individuals, and this mediates a person’s susceptibility to both infection and allergic reaction. At the same time, however, it was argued that GDP can be generated over a wide range of parameter values, meaning that successful GDP defense does not rely on a unique and finely tuned set of kinetic rate constants. In what follows, we consider the flexibility of the GDP mechanism in terms of parameter values.

Table 1 outlines the various parameters (with units) that are used in the GDP model. To test the viability of the GDP mechanism over a range of parameter values, we used the arguments from the previous sections to establish rate constant relationships necessary for effective GDP. Then, within the bounds of these relationships, we tested randomly generated parameter combinations in order to determine the fraction of parameter sets that could successfully accomplish GDP. With respect to GDP success, we note that three criteria are required. First, the GDP parameters must induce immune activation across the entire range of pathogen growth rates that an individual is likely to encounter over his lifetime. Second, the GDP parameters must induce tolerance in response to constant or decreasing antigen loads. Third, the GDP parameters must induce tolerance in response to growth rates that are higher than those expected from commonly encountered pathogens. (In this case, the rapid antigen rise is taken as representing noise or the injection of an antigen into the bloodstream, rather than pathogen growth itself). The third column in Table 1 sets out the parameter ranges and/or relationship bounds used in our simulations.

Table 1 Parameter Values

| Parameter | Unit | Range |
| --- | --- | --- |
| *α_r_* | cells^4^time^-1^volume^-1^ | 1 – 100 |
| *α_17_* | cells^4^time^-1^volume^-1^ | *ρ_α_α_r_* < *α_17_* < *α_r_* |
| *β_r_* | cells^3^time^-1^volume^-1^ | *ρ_αβl_α_r_* < *β_r_* < *ρ_αβh_α_r_* |
| *β_17_* | cells^3^time^-1^volume^-1^ | *ρ_β_β_r_* < *β_17_* < *β_r_* |
| *μ_r_* | time^-1^volume^-1^ | *β_r_*/*K_r_* < *μ_r_* < *ρ_μβ_β_r_*/*K_r_* |
| *μ_17_* | time^-1^volume^-1^ | *μ_r_* < *μ_17_* < *ρ_μ_μ_r_* |
| *K_r_* | cells^3^ | 1-10 |
| *K_17_* | cells^3^ | *K_r_* < *K_17_ < ρ_K_K_r_* |
| *τ* | time | *ρ_τ_α_r_*(*Ω_17_* + *Ω_r_*)/*K_r_* |
| *γ* | cells⋅volume^-1^ | 1-10 |
| *δ* | cells⋅volume^-1^ | 1 × 10^-10^ |
| *v* | cells⋅volume^-1^ | 1 × 10^-14^ |

For the simulations described below, we chose a range of 1-100 cells^4^time^-1^volume^-1^ for *α_r_* and a range of 1-10 cells^3^ for *K_r_*. As can be seen from equation (2), the quotient of these two parameters, *α_r_/K_r_* , can be taken as the maximal rate of T_reg_ cell proliferation in response to APC stimulation (i.e. the rate prior to significant feedback from Th17 cells). Our selected ranges for *α_r_* and *K_r_* thus give a maximal APC stimulated proliferation rate between 0.1 – 100 cells⋅time^-1^volume^-1^. For *γ* we chose a range of 1-10 cells, meaning that the population of actively presenting APCs is at its ½ maximal value when between 0.5-5 cells⋅volume^-1^ are engaged in antigen presentation to T-cells. We select this range primarily because the number of DC cells is not expected to be large. All of the other parameters are governed by the parameter relationships outlined above. Below, we test the effects of altering the strictness of these parameter relationships on GDP operation.

Figure 11 shows the fraction of parameter sets that activate plotted against the log of the pathogen growth rate for four separate parameter conditions. In the first case (diamonds), we take very tight restrictions, with *ρ_α_* = *ρ_β_* = 0.99, *ρ_μ_* = *ρ_K_* = 1.01, and *ρ_μβ_* = 1.05. This implies a difference of up to 1% in stimulation and turnover rates between T_reg_ and Th17 cells, and a difference of up to 5% between (maximal) positive feedback rates and death rates of T_reg_ cells. In the second case (circles), we loosen the restrictions using *ρ_α_* = *ρ_β_* = 0.98, *ρ_μ_* = *ρ_K_* = 1.02, and *ρ_μβ_* = 1.10, which implies a difference of up to 2% in stimulation and turnover rates between T_reg_ and Th17 cells and a difference of up to 10% between (maximal) positive feedback rates and death rates of T_reg_ cells. For the third scenario (triangles), we loosen the restrictions even further, using *ρ_α_* = *ρ_β_* = 0.95, *ρ_μ_* = *ρ_K_* = 1.05, and *ρ_μβ_* = 1.20, while in the fourth scenario (crosses) we use the loosest restrictions, with *ρ_α_* = *ρ_β_* = 0.90, *ρ_μ_* = *ρ_K_* = 1.10, and *ρ_μβ_* = 1.40. In all cases we consider *ρ_αβl_* = 0.10 and *ρ_αβh_* = 0.50, which means that T-cell expansion due to positive feedback is a fraction of T-cell expansion due to APC stimulation. Similarly, for all scenarios we use 30 < *ρ_τ_* < 100, which forces the first term in equation (5) to be significantly larger than the third term, ensuring that real time information regarding the instantaneous change in the antigen load is weighted substantially higher than past information regarding the state of the immune system.

From Figure 11 it is clear that even for randomly selected parameter sets, GDP can effectively detect the presence of pathogens exhibiting a wide range of growth rates. As would be expected, the least sensitive immune systems are the ones with the loosest parameter restrictions. Even for these systems, however, greater than 90% of the parameter sets detect the presence of pathogens over growth rates ranging from *r* = 0.1-5 pathogens⋅time^-1^volume^-1^. This corresponds to a high likelihood of defense against any organism with a doubling period between 0.1 and 7 time units. At the other extreme, greater than 90% of the parameter sets for the most sensitive immune systems (strictest parameter restrictions) detect the presence of pathogens over growth rates ranging from *r* = 0.1-50 pathogens⋅time^-1^volume^-1^. This corresponds to a high likelihood of defense against any organisms with a doubling period between 0.01 and 7 time units.

In the main text, we used *r* = 0.035 for the leprosy bacterium. The reason that this value was selected for the growth rate of leprosy was that it lies on the border of being detectable by most immune systems (53% of our least sensitive immune systems and 69% of our most sensitive immune systems can detect it). Assuming that leprosy does indeed have a 2 day doubling period, *r* = 0.035 implies that the time unit in our simulation is 144 minutes. In other words, over 90% of our least sensitive immune systems should be able to detect pathogens with doubling periods between 14.4 minutes and 17 hours, while over 90% of our most sensitive immune systems should be able to detect pathogens with doubling periods between 1.4 minutes and 17 hours. Naturally, we do not know for sure that the replication rate of leprosy falls right on the border of being detectable by GDP, thus these estimates for immune detection capabilities are relatively rough, and would vary if the growth rate of leprosy was actually more or less likely to be detected by GDP than we have assumed for the sake of parameterization.

In the simulations used for the above analysis, we did not add noise to the antigenic signal. Therefore, to illustrate the effects of noise on our GDP prediction, we repeated our simulations on the two most sensitive scenarios (*ρ_α_* = *ρ_β_* = 0.99, *ρ_μ_* = *ρ_K_* = 1.01, and *ρ_μβ_* = 1.05 and *ρ_α_* = *ρ_β_* = 0.98, *ρ_μ_* = *ρ_K_* = 1.02, and *ρ_μβ_* = 1.10), this time adding noise to the pathogen signal. The results of these simulations are shown in Figures 12a. and 12b. respectively. As would be predicted, the *ρ_α_* = *ρ_β_* = 0.99, *ρ_μ_* = *ρ_K_* = 1.01 immune systems were the most significantly influenced by the noise, exhibiting a higher probability of activation at both low and high replication rates and a lower probability of activation at intermediate replication rates. In the *ρ_α_* = *ρ_β_* = 0.98, *ρ_μ_* = *ρ_K_* = 1.02, and *ρ_μβ_* = 1.10 immune systems, the effects of noise were noticeably smaller. This analysis reiterates the trade-off between sensitivity and robustness that we have already highlighted in previous sections. In particular, while the most sensitive immune systems exhibit pathogen detection over a wider range of pathogen growth rates, they are also more likely to activate in response to a non-threatening antigen and and develop tolerance in response to a pathogen when the antigenic signal itself is relatively noisy.

**E. Future Experiments**. Although the general regulatory network used to develop the GDP model is based on experiment, many of the parameters that appear in the GDP equations remain largely uncharacterized. Therefore, while the overall GDP requirements including positive and negative feedback between T_reg_ and Th17 cells, T_reg_ permission (via TGF-β) of Th17 development and delayed T_reg_ maturation are all supported by empirical studies, it remains difficult to fully parameterize the model for specific vertebrate immune systems (eg. mouse, human). This difficulty with parameterization is somewhat alleviated by our demonstration that effective GDP decision-making is not particularly sensitive to parameter selection. That said, we strongly encourage experimental work that could elucidate the relationships between the various kinetic parameters in our model. Ideally, full characterization of the GDP model would entail measuring the real-time proliferation, turnover and accumulation rates of T_reg_ and Th17 cells in a tissue and context (infection, allergy, autoimmunity, etc) specific manner. Since this would a major experimental feat, we suggest several less complete characterization steps that could be taken more easily with currently available empirical methods.

Recently, Negrin et al have developed a transgenic gfp-luc expressing murine line from which it is possible to obtain any cell type for transplantation studies(1). Purifying T_reg_ and Th17 cells from transgenic donor mice and separately introducing them into either pathogen infected syngeneic or else allogeneic recipients would help to establish the relative abilities of these two T-cell populations to proliferate and persist in response to antigen stimulation. Moreover, bioluminescent imaging would allow proliferation and persistence to be monitored in a tissue specific manner. A faster proliferation rate or a slower death rate associated with T_reg_ cells when compared to Th17 cells would help to support the GDP mechanism.

Unfortunately, the gfp-luc expressing murine line would be less useful in terms of elucidating relative maturation rates of T_reg_ and Th17 cells from naïve T-cell precursors. Therefore, in order to measure relative rates of proliferation from naïve T-cells, an alternative experimental approach would be necessary. One option is BrDU labeling and flow cytometry, although to obtain useful data, a large number of early time-points would need to be considered, thus this type of study would be labor intensive. Nevertheless, a delayed maturation but higher proliferation rate of T_reg_ cells when compared to Th17 cells would again help to support the GDP mechanism.

Although characterization of the kinetic rate constants in our model would be extremely useful, an alternative approach to testing the model would be to examine some of the model predictions. It would be interesting, for instance, to experiment with the effects of varying antigen dose over time in terms of immune system behavior. If, for instance, the immune system is subjected to increasing doses of antigen over time, then according to the GDP model, this should tend to activate the immune system. In contrast, if the immune system is subjected to decreasing doses of antigen over time, then according to the GDP model, this should tend to induce peripheral tolerance. Certainly, the timing of the antigen doses must be carefully chosen in order to avoid significant decay of the antigen signal between antigen administration events. To avoid this problem, different controlled antigen release mechanisms could also be employed to study the effects of antigen kinetics on immune system activation. In particular, the sustained release of antigens from poly(lactic-co-glycolic acid) microparticles has already been proven more effective than antigen alone with respect to eliciting an immune response(2, 3), and this is in keeping with GDP predictions (ie a fast injection of antigen into the blood followed by subsequent antigen decay should induce tolerance while a slower increase in the antigen load should trigger immune activation). Obviously, any tests of the GDP model that involve experimentation with antigen kinetics should be done using antigens that are not recognized by immune mechanisms other than GDP.

References

1. Negrin RS & Contag CH (2006) In vivo imaging using bioluminescence: a tool for probing graft-versus-host disease. *Nat Rev Immunol* 6(6):484-490.

2. Jiang W, Gupta RK, Deshpande MC, & Schwendeman SP (2005) Biodegradable poly(lactic-co-glycolic acid) microparticles for injectable delivery of vaccine antigens. *Advanced Drug Delivery Reviews* 57(3):391-410.

3. Sah H, Toddywala R, & Chien YW (1995) Continuous release of proteins from biodegradable microcapsules and in vivo evaluation of their potential as a vaccine adjuvant. *Journal of Controlled Release* 35(2-3):137-144.

Figures

**Fig. 1.** Phase diagram for immune regulation as a function of $\alpha_{r}/K_{r}:\alpha_{17}/K_{17}$. The parameter set used to generate Figure 1 was: α_17_ = 15.0, β_r_ = β_17_ = 1, μ_r_ = μ_17_ = 1.0, K_r_ = K_17_ = 1.1, γ =1.0, τ = 1, δ = 1 × 10^-10^, *v* = 1 × 10^-14^, p = 3, κ =1000 and α_r_ = 15.01-22.5.

**Fig. 2**. Phase diagram for immune regulation as a function of $\beta_{r}/K_{r}:\beta_{17}/K_{17}$. The parameter set used to generate Figure 2 was α_r_ = α_17_ = 15.0, β_17_ = 1.0, μ_r_ = μ_17_ = 1.0, K_r_ = K_17_ = 1.1, γ =1.0, τ = 1, δ = 1 × 10^-10^, *v* = 1 × 10^-14^, p = 3, κ =1000 and β_r_ = 1.01-3.00.

**Fig. 3**. Phase diagram for immune regulation as a function of $\mu_{r}:\mu_{17}$. The parameter set used to generate Figure 3 was α_r_ = α_17_ = 15.0, β_i_ = β_17_ = 1.0, μ_17_ = 1.0, K_r_ = K_17_ = 1.1, γ =1.0, τ = 1, δ = 1 × 10^-10^, *v* = 1 × 10^-14^, p = 3, κ =1000 and μ_r_ = 0.001-1.

**Fig. 4**. Phase diagrams for immune regulation as a function of τ. Regions of activation and tolerance are shown at both the low replication rate detection limit (a) and the high replication rate detection limit (b), The parameter set used to generate Figure 4 was α_r_ = 15.1, α_17_ = 15.0, β_i_ = β_17_ = 1.0, μ_17_ = μ_r_ = 1.0, K_r_ = K_17_ = 1.1, γ =1.0, δ = 1 × 10^-10^, *v* = 1 × 10^-14^, p = 3, κ =1000 and τ = 1-5.

**Fig. 5**. Phase diagrams for immune regulation as a function of α_r_ = α_17_ + 0.1. Regions of activation and tolerance are shown at both the low replication rate detection limit (a) and the high replication rate detection limit (b), The parameter set used to generate Figure 4 was β_i_ = β_17_ = 1.0, μ_17_ = μ_r_ = 1.0, K_r_ = K_17_ = 1.1, γ =1.0, τ = 1, δ = 1 × 10^-10^, *v* = 1 × 10^-14^, p = 3, κ =1000 and α_r_ = 15.1-20.1, α_17_ = 15.0-20.0.

**Fig. 6**. Phase diagrams for immune regulation as a function of Ω_17_ + Ω*_r_*. Regions of activation and tolerance are shown at both the low replication rate detection limit (a) and the high replication rate detection limit (b), The parameter set used to generate Figure 4 was α_r_ = 15.1, α_17_ = 15.0, μ_17_ = μ_r_ = 1.0, K_r_ = K_17_ = 1.1, γ =1.0, τ = 1, δ = 1 × 10^-10^, *v* = 1 × 10^-14^, p = 3, κ =1000 and β_i_ = β_17_ = 0 – 1.09,

**Fig. 7** Steady states of equation (4) when *d*(*t – τ*) = 0.015. Stable manifolds are shown with solid lines, while the unstable manifold is shown with a dotted line. The vertical grey line marks the boundary along which *d*(*t*) = *d*(*t – τ*) (constant signal), while the horizontal grey line marks the boundary along which *Δ* = 0 (naïve system/no immune decision). Parameter values used for this simulation are *α_r_=*15.1, *α*_17_*=*15.0, *β_r_* =*β*_17_ =1.0,*μ_r_* =1.0,*μ*_17_ =1.1,*δ=*1×10^-10^,*v* = 0, *τ*=1.0, and *K_r_*=*K*_17_ = 1.1.

**Fig. 8** The steady-states of equation (4). Parameter values used for this simulation are identical to those in Figure 1. The arrow shows how an activated immune system (Δ > 0) starting from (×) must experience a *significant* decrease in APC cells (ie. a significant decrease in the antigen population) in order to revert to the suppressed steady-state (Δ < 0).

**Fig. 9** Stability diagram for equation (4). Parameter values used for this simulation are identical to those in Figure 1.

**Fig. 10.**  Phase diagram representing the behavior of the GDP mechanism in terms of the antigen growth factor over τ.

**Fig. 11.**  Percentage of randomly selected immune parameter sets that activate as a function of pathogen growth rate, *r.* For this figure we use the parameter ranges and relationships outlined in Table 1 of the SI. The relationship restrictions applied are as follows: *ρ_α_* = *ρ_β_* = 0.99, *ρ_μ_* = *ρ_K_* = 1.01, and *ρ_μβ_* = 1.05 (diamonds), *ρ_α_* = *ρ_β_* = 0.98, *ρ_μ_* = *ρ_K_* = 1.02, and *ρ_μβ_* = 1.10 (circles), *ρ_α_* = *ρ_β_* = 0.95, *ρ_μ_* = *ρ_K_* = 1.05, and *ρ_μβ_* = 1.20 (triangles), *ρ_α_* = *ρ_β_* = 0.90, *ρ_μ_* = *ρ_K_* = 1.10, and *ρ_μβ_* = 1.40 (crosses). In all cases we assume 30 < *ρ_τ_* < 100 and an antigenic signal without noise.

**Fig. 12.**  Percentage of randomly selected immune parameter sets that activate as a function of pathogen growth rate, *r.* As in Figure 11, we use the parameter ranges and relationships outlined in Table 1 of the SI. The relationship restrictions applied are as follows: *ρ_α_* = *ρ_β_* = 0.99, *ρ_μ_* = *ρ_K_* = 1.01, *ρ_μβ_* = 1.05 and 30 < *ρ_τ_* < 100 (a), *ρ_α_* = *ρ_β_* = 0.98, *ρ_μ_* = *ρ_K_* = 1.02, *ρ_μβ_* = 1.10 and 30 < *ρ_τ_* < 100 (b). Closed circles represent simulations without noise and the open circles represent simulations with high frequency noise added to the antigenic signal.
